# Case report: A case of giant accessory hepatic lobe torsion combined with left hepatic vein branch thrombosis in a child

**DOI:** 10.3389/fped.2022.970876

**Published:** 2022-09-26

**Authors:** Xiangang Xiong, Yonghua Lou, Teng Zhou, Zebing Zheng, Yuanmei Liu, Rui Liu, Kaizhi Zhang, Yuan Gong, Chengyan Tang, Zhu Jin

**Affiliations:** ^1^Department of Pediatric Surgery, Affiliated Hospital of Zunyi Medical University, Zunyi, China; ^2^Department of Pediatric Surgery, Guizhou Children’s Hospital, Zunyi, China; ^3^School of Preclinical Medicine, Zunyi Medical University, Zunyi, China

**Keywords:** accessory hepatic lobe, accessory hepatic lobe torsion, left hepatic vein branch thrombosis, child, liver

## Abstract

The accessory hepatic lobe (AHL) is a rare congenital malformation of the hepatic tissue, among which the giant AHL is the rarest in children. Patients without complications are usually asymptomatic, and most auxiliary examinations cannot provide a definitive preoperative diagnosis. Surgical procedure is the only recommended management for patients who suffered from the complications of AHL. We report the case of a rare pediatric giant AHL torsion combined with left hepatic vein branch thrombosis which was successfully treated by laparoscopic lobectomy followed by excision of AHL.

## Introduction

An accessory hepatic lobe (AHL) is a rare congenital malformation of the liver, and even rarer in the case of AHL with a stalk. When it is large and lacks anatomical fixation by hepatic ligaments, it is susceptible to rotation or even torsion, which can lead to ischemic infarction (1, 2). It is universally acknowledged that any symptomatic giant AHL in children is the rarest. Azmy (3) first described a neonatal case of Beckwith–Wiedemann syndrome combined with an accessory hepatic lobe torsion in 1980. Among 23 reported cases of pediatric AHL, only 3 have undergone laparoscopic resection of twisted AHL (4–6). We report the case of a rare pediatric giant AHL torsion combined with left hepatic vein branch thrombosis which was successfully treated by laparoscopic lobectomy followed by excision of AHL.

## Case

**FIGURE 1:**
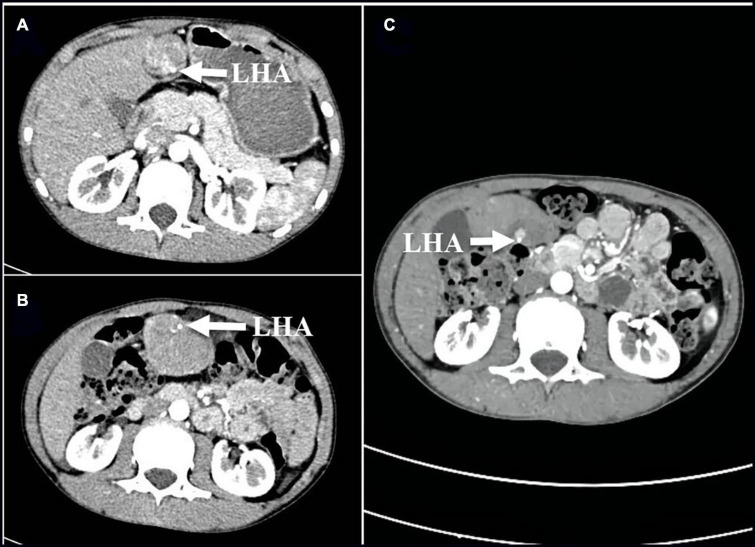
**(A,B)** Cross-sectional images of the first enhanced CT scan before admission on 25 April 2021 show 180° torsion of the left hepatic artery (arrow); **(C)** On 27 April 2021, compared with the previous CT scan, the enhanced CT scans repeated after the disappearance of abdominal pain symptoms suggested that the twisted hepatic artery has reset itself (arrows). LHA, left hepatic artery; CT, computed tomography.

**FIGURE 2:**
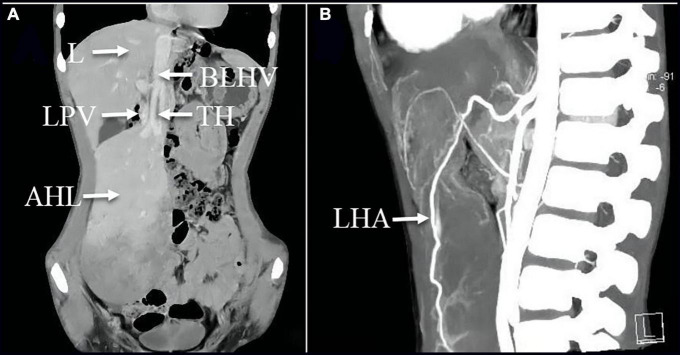
**(A)** Coronal image of the enhanced CT scan on 27 April 2021 revealed the mass was visible as non-uniform reinforcement in the inferior portion; **(A,B)** CTA images illustrated that the left hepatic artery, the branch of the left hepatic vein, and the left branch of the portal vein were observed within the mass, but multiple filling defects are recognized inside the branch of the left hepatic vein. AHL, accessory hepatic lobe; TH, thrombosis; L, liver; LPV, left hepatic portal vein; BLHV, the branch of the left hepatic vein; LHA, left hepatic artery.

A 12-year-old male child was admitted to the emergency room with abdominal pain; the child presented with persistent vague pain in the right abdomen without cause, accompanied by vomiting a gastric content. Vital signs were stable, and a tough mass about 19 cm × 10 cm × 6 cm in size detected in the right lower and middle abdomen was movable and obviously painful. Laboratory tests showed only mild elevation of alanine aminotransferase and portal aminotransferase. Abdominal Doppler ultrasound revealed a hypoechoic mass measuring approximately 146 × 44 mm connected to the liver was identified in the left outer lobe of the liver. On 25 April 2021, the abdominal enhancement computed tomography (CT) was performed in the Emergency Department of the Affiliated Hospital of Zunyi Medical University, suggesting a large mass of approximately 170 × 95 × 40 mm in size protruding into the pelvis and attached to the left lobe of the liver by a twisted stalk (Figures 1A,B). The abdominal pain and vomiting disappeared after the child was hospitalized. On 27 April 2021, a re-examination of the abdomen by contrast-enhanced CT revealed that the twisted AHL had spontaneously reverted to its location (Figure 1C). The AHL was visible as non-uniform reinforcement in the inferior portion, with density similar to the liver parenchyma (Figure 2A). The computed tomography angiography (CTA) illustrated that the left hepatic artery, the left hepatic vein branch, and the left branch of the portal vein were observed within the mass, but multiple filling defects were recognized inside the branch of the left hepatic vein (Figures 2A,B).

**FIGURE 3:**
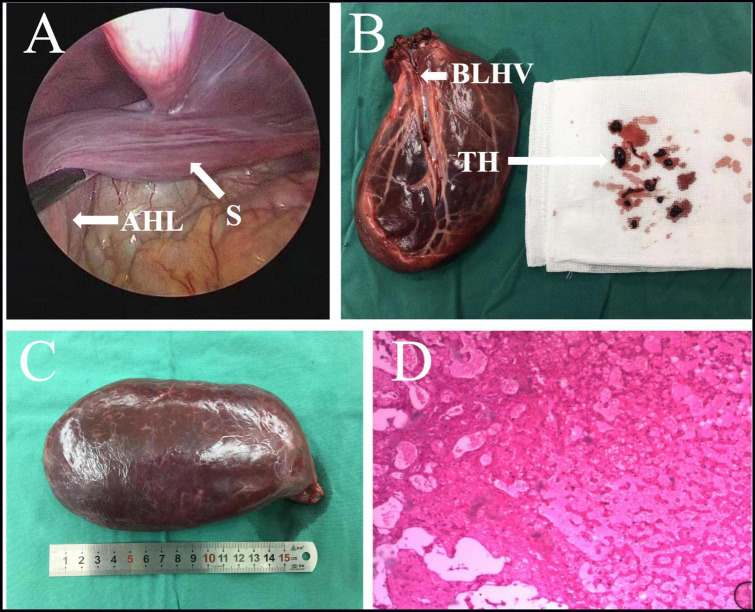
**(A)** Intraoperative images suggested the AHL has repositioned itself, with only a stalk of approximately 2 cm in diameter liver tissue forming an attachment to the left liver. **(B,C)** Excision of large AHL with thrombosis of a branch of the left hepatic vein. **(D)** The histopathology result demonstrated the irregular structure of the hepatic lobules, dilated hepatic sinusoids, and disordered vascular distribution in the portal area and interlobular. AHL, accessory hepatic lobe; S, stalk; TH, thrombosis; BLHV, branch of the left hepatic vein.

The patient was placed in the supine position, and laparoscopy was performed using a 30°camera (STORZ). A total of four 5 mm trocars were placed at the following port sites: an infraumbilical, a left upper quadrant, and two right upper quadrant ports. During the laparoscopic procedure, it was diagnosed that a giant AHL of an estimated size of 18 × 10 × 6 cm was visible at the left side of the hepatic round ligament, with only a 2 cm in diameter liver tissue forming a stalk adjacent to the left liver, through which the branch of the left hepatic vein, the left branch of the portal vein, the left hepatic artery, and the bile duct were passing through (Figure 3A). The twisted stalk had repositioned itself. Partial infarction had occurred in the lower part of the giant AHL entering the pelvis, with an excellent flow of blood in the upper part. Careful dissection by using a 5-mm harmonic scalpel (Ethicon) was performed on blood vessels and bile duct ligated with the use of Hem-o-lock clips (Weck) through the stalk, and ligation of the stalk facilitated resection of the giant AHL. However, owing to the large size of the AHL, an incision of 6 cm in length was made in the midline of the abdomen to remove the AHL, weighing 552 g, from the abdominal cavity. The branch of the left hepatic vein was dissected *in vitro*, and a large amount of thrombosis was obtained (Figures 3B,C). The histopathology result demonstrated the irregular structure of the hepatic lobules, dilated hepatic sinusoids, and disordered vascular distribution in the portal area and interlobular (Figure 3D). The prognosis was satisfactory at the postoperative 2-month follow-up.

## Discussion

The exact pathogenesis of AHL remains unknown. One theory states that it is associated with a malformation of the caudal foregut of the endoderm and an abnormal division of the tissue buds during the 3rd week of gestation (7). AHL is categorized on the basis of size and its attachment to the liver: (1) large AHL (weight >30 g), (2) small AHL (weight <30 g), (3) ectopic AHL without hepatic attachment, and (4) microscopic AHL in the gallbladder wall (8). Among the 23 pediatric AHL cases reported in the literature, the age ranged from 1 day to 15 years, the male to female ratio was 11:12, and laparoscopic resection of AHL torsion has been reported (4–6). AHL was typically an incidental finding on radiographic imaging or autopsy, and most cases were also associated with persistent defects of anterior abdominal wall (4, 5). Corbitt et al. (4) reported that 41% of children with AHL are associated with abdominal wall defects, including umbilical bulge, Beckwith–Wiedemann syndrome, umbilical hernia, and cloacal exstrophy, stemming from abnormal development of the AHL that may obstruct the closure of the umbilical ring (4). A torsion of the large AHL may result in venous thrombosis, but such a large venous thrombosis has not been observed in the reported pediatric cases. Another report suggests that the risk of malignancy in AHLs is higher than in healthy liver tissue because of impaired blood supply and bile duct drainage (9, 10). In some instances, on imaging, it is a possibility to consider the AHL as a tumor or abscess (11). The patient was diagnosed with giant AHL which is extremely rare in children.

Patients with uncomplicated AHL are usually asymptomatic. Large AHL torsion with strangulation of the supplying vessels often resulting in severe hepatic ischemia is possibly caused by strenuous exercise or trauma (12). In that case, most patients are presented with sudden severe right-sided abdominal pain, probably accompanied by nausea or vomiting. As for palpation, an enlarged tender mass might be detected (5, 13). The AHL should not be reversed before ligation to prevent dislodgement of the thrombus (2). AHL torsion could be misdiagnosed as any other acute abdominal disease or intra-abdominal tumor. In this case, the giant AHL is attached to the left lobe of the liver through a stalk formed by normal liver tissue, with vague pain in the right mid-lower abdomen and lacked a history of trauma. Laparoscopic exploration revealed infarction of the lower part of giant AHL, but the twisted giant AHL had repositioned itself.

Most reports do not provide a definitive preoperative diagnosis. Ultrasonography, CT, and magnetic resonance imaging (MRI) are recommended for the diagnosis of large diameter AHL, while the auxiliary examinations could not detect the small diameter AHL. Some researchers proved that ultrasonography had a limited diagnostic value in the diagnosis of AHL (6). MRI indicates superior diagnostic value to CT, as the vascular system extending from the healthy liver through the stalk into the giant AHL is more likely to be detected by dynamic contrast-enhanced MRI (2). However, it has been suggested that for complete stalk torsion, a contrast enhancement CT scan, especially CTA, is more useful than ultrasonography for diagnosis (14). Ultrasonography, plain CT scan, and MRI have also been reported to be non-specific for diagnostic imaging findings in pediatric AHL patients (5). Hepatic infarcts often showed as hypoechoic, non-vascular areas on ultrasonography (15). The typical AHL demonstrated on CT is a mass with a narrow base or broad bottom attached to the healthy liver, with the Hounsfield unit consistent with healthy liver tissue (16). This case was in accordance with the CT presentation of the AHL, and the CTA was applied subsequently for further evaluation of the vascular system. CTA revealed thrombosis in a left hepatic vein branch. However, ultrasonography only indicates hypoechoic mass and was of limited diagnostic value in this case. CT is of better diagnostic value than ultrasound for AHL.

Most patients with an AHL will not demand medical treatment. A close follow-up would be advisable in asymptomatic cases. Surgical management is only necessary for those individuals suffering from complications such as torsion, rupture, and pedunculated AHL. Prompt management and emergency excision or laparoscopy should be recommended in cases of suspected AHL torsion (6, 17). Even among individuals presenting with complications, most do well following surgery (4–6). As this disease is relatively rare, torsion of the giant pedunculated AHL was already evident at the onset of abdominal pain in this child, but the diagnosis on early imaging failed and emergent operation was not performed. During examination, the torsion has reset itself and the abdominal pain disappeared. The child eventually underwent laparoscopy-assisted resection of the AHL.

## Conclusion

This case is the first report of a rare stalk giant AHL torsion along with the left hepatic vein branch thrombosis. The AHL was resected by laparoscopic-assisted surgery, thus minimizing disruption to the abdominal cavity. When it is large and lacks anatomical fixation by hepatic ligaments, it is susceptible to rotation or even torsion, which can lead to ischemic infarction. The AHL needs to be differentiated by pediatricians from any other abdominal disease. We recommend laparoscopy as the first-choice treatment modality for complicated AHL.

## Data availability statement

The original contributions presented in this study are included in the article/supplementary material, further inquiries can be directed to the corresponding author.

## Ethics statement

Written informed consent was obtained from the minor(s)’ legal guardian/next of kin for the publication of any potentially identifiable images or data included in this article.

## Author contributions

XX and YL wrote the first draft of the manuscript. ZJ wrote sections of the manuscript. All authors contributed to manuscript revision, read, and approved the submitted version.

## Abbreviations

AHL: accessory hepatic lobe
TH: thrombosis
L: liver
LPV: left portal vein
BLHV: the branch of the left hepatic vein
S: stalk
LHA: left hepatic artery
CT: computed tomography
MRI: magnetic resonance imaging.
